# The Impact of Seasonal Changes on Thyroxine and Thyroid-Stimulating Hormone in Newborns

**DOI:** 10.3390/ijns7010008

**Published:** 2021-02-03

**Authors:** Rebecca McMahon, Lenore DeMartino, Mycroft Sowizral, Diana Powers, Melissa Tracy, Michele Caggana, Norma P. Tavakoli

**Affiliations:** 1 Wadsworth Center, Division of Genetics, New York State Department of Health, Albany, NY 12208, USA; rebecca.mcmahon@health.ny.gov (R.M.); lenore.demartino@health.ny.gov (L.D.); michele.caggana@health.ny.gov (M.C.); 2 Scientific Core, Wadsworth Center, New York State Department of Health, Albany, NY 12208, USA; mycroft.sowizral@health.ny.gov; 3 Mathematics Department, West Virginia University Institute of Technology, Beckley, WV 25801, USA; piccpowers@gmail.com; 4 Department of Epidemiology and Biostatistics, State University of New York, Rensselaer, NY 12144, USA; mtracy@albany.edu; 5 Department of Biomedical Sciences, State University of New York, Albany, NY 12208, USA

**Keywords:** newborn screening, congenital hypothyroidism, thyroxine, thyroid stimulating hormone, seasonal

## Abstract

Newborn screening for congenital hypothyroidism (CH) is performed by measuring the concentration of thyroxine (T4) and/or thyroid-stimulating hormone (TSH) in dried blood spots. Unfortunately, the levels of T4 and TSH vary due to multiple factors, and therefore the false-positive rate for the test is a challenge. We analyzed screening data from 2008 to 2017 to determine the effect of seasonal changes and manufacturer kit lot changes on T4 and TSH values and on numbers of infants referred. Over a 10-year period, we screened 2.4 million infants using commercially available fluoroimmunoassays to measure T4 and TSH concentrations in dried blood spots. During colder months, daily mean T4 and TSH values were higher and referral rates and false-positive rates were higher. However, there was no significant difference between the number of confirmed CH cases. Furthermore, in rare instances, we observed differences in T4 daily mean values during the 10-year period when manufacturer kit lot changes were made. Seasonal temperature variations influence measured T4 and TSH values and consequently lower the positive predictive value for CH testing in colder months. Newborn screening (NBS) programs should be aware that manufacturer kit lot changes may also influence T4 values.

## 1. Introduction

Newborn screening (NBS) for congenital hypothyroidism (CH) is performed routinely in most of the developed world, where it has led to the near elimination of intellectual disability caused by this common condition. Early detection is important as treatment should be initiated as early as possible, preferably within the first two weeks of life [1]. NBS laboratories use different algorithms for screening for CH. In recent years, the majority of programs perform a first-tier thyroid-stimulating hormone (TSH) screen, sometimes in combination with a second-tier thyroxine (T4) screen [2]. A minority of programs perform a first-tier T4 screen followed by a second-tier TSH screen and less frequently in combination with a TSH screen. [2]. The difficulty with measuring TSH and T4 is that many factors affect the concentration of these analytes in newborns. Notably, due to the stress of birth and exposure of the infant to the cold environment, TSH is elevated during the first 0–48 h after birth and T4 is low [3,4,5]. Additionally, low birth weight (LBW) and premature infants have low T4 levels at birth, which normalize with advancing neonatal age [6,7]. The immaturity of the hypothalamic–pituitary axis leads to lower preterm serum T4 and free T4 concentrations, and a qualitatively similar but quantitatively lower TSH surge comparable to that of term infants [5,8,9]. Furthermore, preterm infants frequently have thyroxine-binding globulin (TBG) deficiency due to immature liver function and undernutrition [10], which leads to lower serum T4 concentrations. In recent years, there has been a dramatic increase in the survival of very LBW babies, who in addition to having low T4 at birth also have a higher incidence of transient hypothyroidism than full-term infants [11]. These factors lead to frequent false-positive CH results, especially when specimens are collected too soon after birth and in premature or LBW infants. To prevent unnecessary referrals, many NBS programs have higher cut-offs for TSH when the specimen is collected within the first 24 h after birth. Additionally, age-related cut-offs have been recommended [12,13]. Furthermore, it is recommended that a second specimen be collected from pre-term and LBW infants at 2–4 weeks of life and/or at discharge from the neonatal intensive care unit (NICU) [1,14,15]. We investigated other factors that affect T4 and TSH concentrations to determine whether they contribute to false-positive CH rates, which cause anxiety for parents and create additional work and expense for the health care system.

## 2. Materials and Methods

From 2008 to 2017, the New York State (NYS) NBS program screened 2.4 million babies for CH. In NYS, ideally, a blood specimen is collected via a heel stick from all newborns on a specimen collection card (Eastern Business Forms, Greenville, SC) 24–48 h after birth and sent to the NBS program with accompanying mother and infant demographic information. Throughout the period of the study, the program required that specimens be shipped overnight at ambient temperature. During accessioning, specimens were manually checked for suitability for testing. Unsuitable specimens such as those with serum rings, blood clots or specimens of insufficient quantity were not tested prior to 7 November, 2016. Instead, a repeat specimen was requested. After this date, many of these specimens were considered “suboptimal” specimens, were tested, and an additional specimen was also requested.

**Figure 1:**
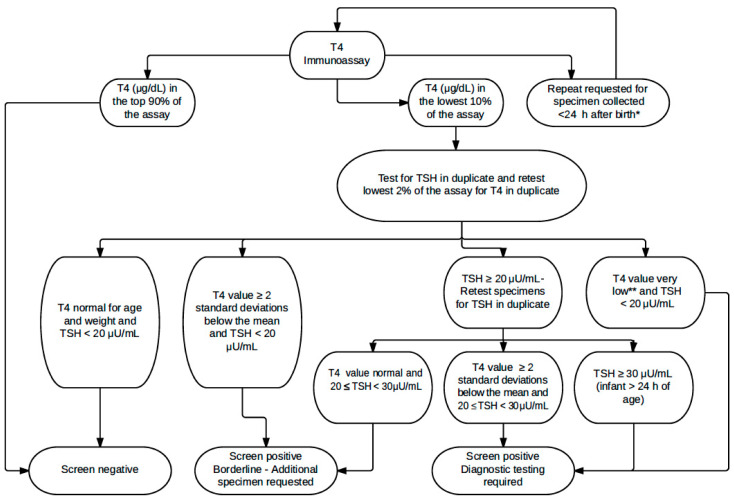
Congenital hypothyroidism testing algorithm in NYS during this study {Adapted from [16]. Copyright (2018), Bioscientifica, Ltd.}. * A repeat specimen is requested when a specimen collected on day of birth (DOB) is received but the specimen is nevertheless tested. ** T4 value very low is 2.5 μg/dL for an initial specimen in a ≥2500 g baby collected at <14 days of age, although the value varies based on birth weight, age at specimen collection, and repeat status. If the TSH value of a DOB specimen is ≥150 μU/mL, the infant is immediately referred to a paediatrician for investigation of probable congenital hypothyroidism (CH). For a non-DOB specimen, an infant with a TSH value ≥100 μU/mL is immediately referred. T4, thyroxine; TSH, thyroid-stimulating hormone.

Screening for CH was performed using AutoDELFIA Neonatal Thyroxine and hTSH kits (Perkin Elmer, Turku, Finland) as described previously using a first-tier T4 and second-tier TSH algorithm [16] (Figure 1). A 10% cut-off was established for each 2-plate assay, which was composed of two 96-well plates, each containing 87 patient specimens. However, the 10% cut-off was increased if the specimens in the 10% included a large number of specimens from low-birth-weight newborns or repeat specimens. T4 and TSH results are reported in serum equivalent units.

The average daily temperature was obtained from the National Weather Service’s NOWData, http://w2.weather.gov/climate/, for New York City (accessed on 26 April, 2018). From 2008 to 2017, approximately half of New York State (NYS) births occurred in the New York City (NYC) area. Therefore, the average daily temperature of NYC was selected as the state’s average temperature. Graphs of average daily T4 and TSH values and average daily temperatures were constructed for each year. For this study, winter included the months January, February, and March and summer included July, August, and September.

### Statistical Analysis

To assess the relationship between T4 and TSH mean values and temperature, the average daily temperature in NYC was matched to each specimen’s collection date. For each Julian day, all T4 and TSH values collected on that day were averaged. Lastly, the averaged T4 and TSH values were plotted against the mean temperature on that day, and the relationship was modeled using local regression (LOESS). To assess the fit of the LOESS-predicted relationship, the error between each point and its prediction was plotted against temperature. Scatterplots with LOESS lines were created using RStudio Version 1.1.442 (RStudio, Boston, MA, USA).

Basic techniques from time series analysis were used to visualize the seasonality of the T4 and TSH data. The methodology was applied to both daily mean T4 and TSH data. By applying two moving averages with specific window sizes, the T4 and TSH time series were decomposed into their cyclic components, with the goal of isolating the annual cycle based on the following model:

T4(t) or TSH(t) = long-term trend(t) + annual cycle(t) + noise

Differences in the numbers of infants referred, false-positive cases, and confirmed CH during winter and summer were assessed using Chi-square tests and Fisher’s Exact Tests. A *p*-value of <0.05 was considered statistically significant.

## 3. Results

CH screening data for 2.4 million infants were analyzed. Specimen submission was higher in summer and lower in winter, with a difference of approximately 4–8000 each year. Additionally, every year, a higher proportion of specimens were submitted from LBW infants (12.1% vs. 11.6% overall) and from >14-day-old infants (6.0% vs. 5.3%) in the winter.

The minimum mean T4 for the period 2008 to 2017 was 12.2 μg/dL serum (157.0 nmol/L), the maximum was 24.2 μg/dL (311.5 nmol/L), and the mean (median) was 17.5 (17.3) μg/dL (225.3 and 222.7 nmol/L, respectively). The minimum mean TSH for the period 2008 to 2017 was 4.5 μU/mL serum, the maximum was 14.1 μU/mL, and the mean (median) was 8.1 (8.8) μU/mL. There was an outlier with a mean TSH daily value of 18.7 μU/mL on a day with a large proportion of specimens from infants <24 h old.

**Figure 2:**
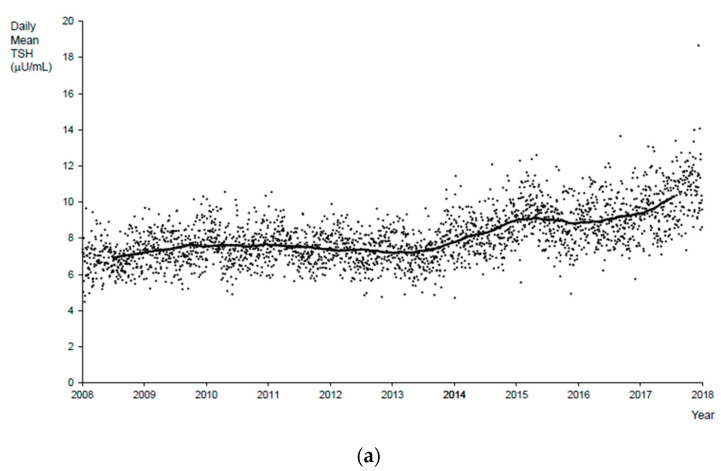

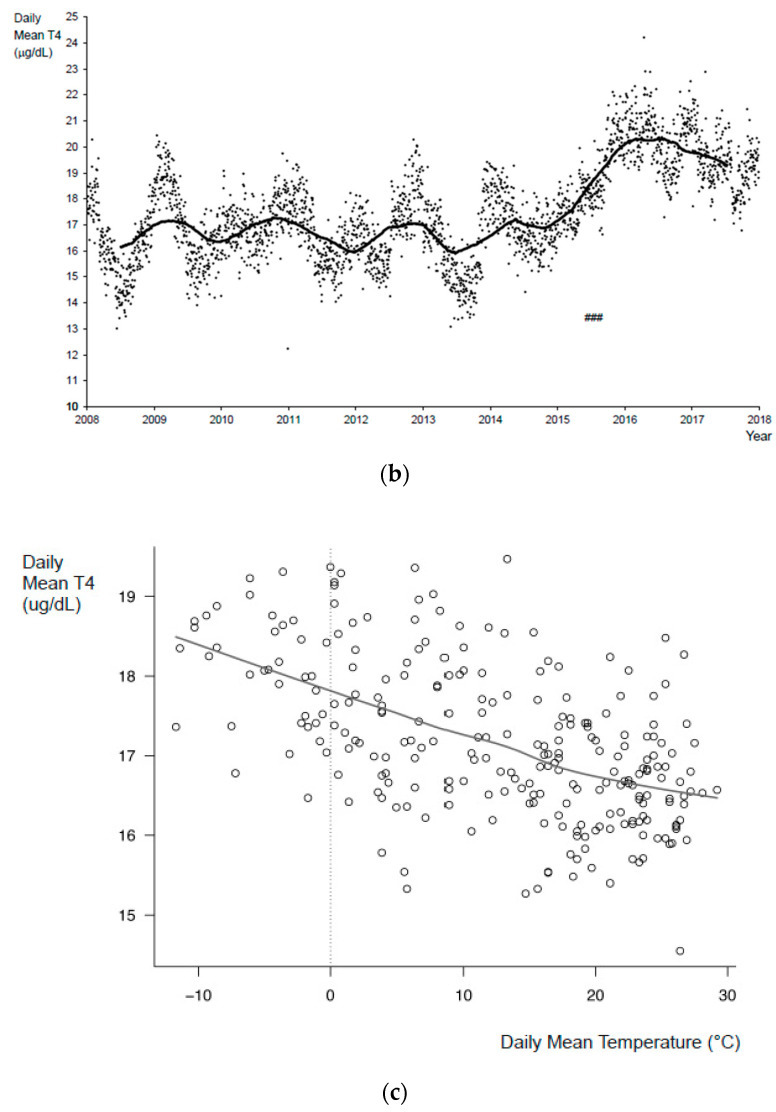
(**a**) Daily mean TSH values for all specimens from 2008 to 2017. The black dots represent daily mean TSH values. The black line represents the long-term trend of daily TSH values. (**b**) Daily mean T4 values for all specimens from 2008 to 2017. The black dots represent daily mean T4 values. The black line represents the long-term trend of daily T4 values. Hashtags represent the kit lot change from 27 April to 9 September, 2015. Note that the T4 levels are higher when the ambient temperature is low, and with increasing temperature, the daily mean T4 levels decrease. (**c**) Daily mean T4 values and daily mean temperature for 2014. The open circles indicate the daily mean values, and the solid line is the LOESS trend. Note that the T4 levels are higher when the ambient temperature is low, and with increasing temperature, the daily mean T4 levels decrease.

Submission of specimens collected from babies <24 h old increased from 1.4% in 2008 to 4.3% in 2017. Because TSH is elevated in specimens collected from babies who are less than 24 h old, when these specimens are included in daily mean values for TSH, they result in higher daily mean values, and this is especially evident from 2014 onwards (Figure 2a). When specimens that were collected at <24 h were excluded from daily mean TSH values, the trendline indicates a slight increase over time, but is much less obvious (Supplemental Figure S1).

Analysis of daily mean T4 values revealed a significant seasonal variation (Figure 2b). During colder months, the daily mean T4 values were higher, and during hot, humid months, the daily mean T4 values were lower. This pattern was clear from 2008 to 2014 but was not apparent in 2015 and was less clear in 2017. Additionally, including or excluding specimens collected <24 h after birth made a smaller difference in daily mean T4 than in daily mean TSH values. The daily mean T4 values were marginally higher when these specimens were excluded.

Plots of daily mean T4 values and the daily mean temperature in NYC were constructed for each year from 2008 to 2017. The trend of the plots for each year looked similar, with mean T4 values decreasing with increasing temperature (Figure 2c represents data from 2014); the corresponding error plot forms a roughly horizontal line at 0 (Supplemental Figure S2), indicating that the assumption that the relationship between temperature and mean T4 value is linear is reasonable. The only exception to the seasonal variation trend was 2015.

Within any one year, the variation seen in daily mean T4 due to seasonal changes alone ranged from approximately 4.9 μg/dL (63.1 nmol/L) in 2014 to approximately 7.3 μg/dL (94.0 nmol/L) in 2008. There was a statistically significant difference in daily mean T4 values between the winter and summer seasons for the years 2008–17 (*p* < 0.0001).

Notwithstanding the seasonal changes, an increase in daily average T4 values was seen during the period of this study (Figure 2b). The increase coincided mainly with one of the total 34 T4 kit lot changes that occurred from 27 April to 9 September, 2015.

The results of the LOESS analysis were confirmed by the time series analysis. Initially, a moving average with a window width of 365 days was applied to the T4 data. The result was the T4 series where cyclic components with periods of 365 days and lower were removed, as the moving average smoothed them, removing them from the original series. This smoothed T4 series contained longer-period cycles of greater than 365 days, the noncyclic long-term trend including breaks in series in mid-2015 and 2017 arising from T4 kit lot changes (Figure 2b).

**Figure 3:**
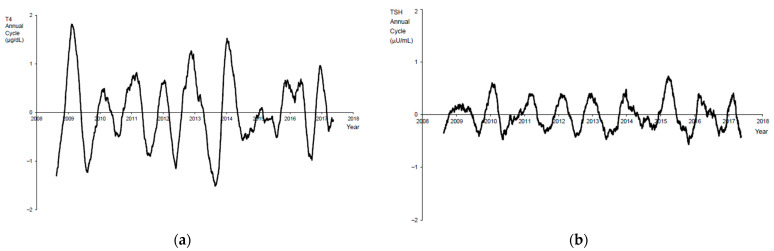
(**a**) Time series analysis isolating the annual cycle component of the T4 series. Note the cyclical seasonal pattern. (**b**) Time series analysis isolating the annual cycle component of the TSH series. Note the cyclical seasonal pattern.

The residual series obtained by subtracting the long-term trend from the original series contained shorter-period cycles of 365 days and below, along with noncyclic noise. Another moving average with a shorter window, 91 days, was applied to the residual series in order to isolate the annual cycle. This second moving average removed any shorter-period cycles of 91 or fewer days, isolating the annual cycle component of the T4 series (Figure 3a).

The same methodology was applied to the TSH data to obtain the long-term trend component of the daily mean TSH (Figure 2a) and its annual cycle (Figure 3b).

Once the deconstruction of the T4 data was completed, the annual cycle from the T4 data (Figure 3a) showed variation of approximately ±1.5 μg/dL each year with clear local maximums in the winter and local minimums in the summer. This represents an annual seasonal variation of approximately ±8%. The T4 annual cycles between mid-2014 and mid-2016 were suppressed because of the mid-2015 series break arising from the T4 kit lot change. Similar analysis of the annual cycle of the TSH data (Figure 3b) showed annual variation of approximately ±0.5 μU/mL with local maximums in the winter and local minimums in the summer. This suggests seasonal variation in the TSH data of approximately ±6%. No series breaks were apparent in the TSH data.

Review of the kit and in-house quality control material indicates that seasonal variations do not affect the performance of these controls whereas kit lot changes do affect the daily mean values of quality control material. Each lot of AutoDELFIA Neonatal T4 kit includes a new lot of quality control material, whereas from 2008 to 2017, the in-house T4 control lot used in our laboratory was only changed once in January 2015. By reviewing the daily values of the quality control material, it is apparent that T4 values were higher after the kit lot change on 27 April, 2015. For example, the one-month mean of the high T4 control prior to 27 April, 2015 was 11.7 μg/dL, whereas the one-month mean after the kit lot change was 12.9 μg/dL, illustrating a 10% increase.

The total number of referrals, CH-affected babies, and false-positives were calculated for the periods January to March and July to September for every year from 2008–17 (Table 1). The number of babies diagnosed with CH and incidence of CH were similar in winter and summer (Table 1). Included in the confirmed cases are 11 central CH cases detected in the winter and 15 central CH cases detected in the summer (*p* = 0.5613). Although the number of confirmed cases was similar, the referral and false-positive rates were higher in winter than in summer (Table 1). The positive predictive value (PPV) of CH testing was lower in winter (14.7%) than in summer (20.6%) (*p* < 0.0001).

**Figure 4:**
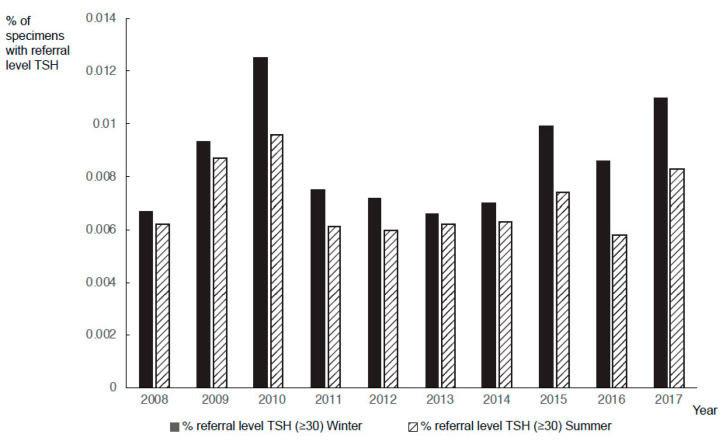
Percentage of specimens with referral-level TSH (≥30 μU/mL) from 2008–17 excluding specimens collected <24 h after birth. Black bars represent percentage of specimens with referral-level TSH values in the winter, and hatched lines represent percentage of specimens with referral level TSH values in the summer.

To investigate the reason for the higher false-positives in winter (1368) vs. summer (1088) (*p* < 0.0001), the referrals were categorized based on the reason they were referred (Figure 1). In the summer, a higher number of false-positive cases were referred because of low T4 and normal TSH (winter: 472 vs. summer: 537) (*p* = 0.4376), although not significantly so. In winter, there were significantly more false-positive cases referred due to elevated TSH (winter: 896 vs. summer: 575) (*p* < 0.0001). The numbers of specimens with borderline TSH (20–29 μU/mL) and referral-level TSH (≥30 μU/mL) were significantly higher during colder months (*p* < 0.0000001). The proportion of specimens with referral-level TSH was higher in the winter than in the summer for every year from 2008 to 2017 irrespective of whether specimens from <24 h infants were included or not (Figure 4).

## 4. Discussion

NBS for CH is complicated because T4 and TSH values are affected by factors including prematurity, birth weight, and age at specimen collection [3,5,15]. Additionally, differences in T4 and TSH values in male and female infants lead to a higher number of false-positive cases amongst male infants [16]. NYS has a humid, continental climate with a large seasonal temperature difference. The summer months are humid and hot, and winter months are cold, sometimes severely so. In this study, we show that seasonal variations affect both T4 and TSH values. Of note, our TSH data do not represent the entire population but a subset of approximately 14%, which had the lowest T4 values. Our data show an increase in the number of cases with elevated TSH during colder months, leading to an increase in the number of referrals. Seasonal changes in daily TSH values correlating with an increase in the false-positive rate has been reported previously in Iowa [17]. In addition, seasonal variations have been reported in other enzymes (e.g., immunoreactive trypsinogen, the marker for cystic fibrosis) and hormones (e.g., 17-hydroxyprogesterone, the marker for congenital adrenal hyperplasia) that are routinely measured in NBS programs [17,18,19].

A cyclical seasonal pattern is observed for both T4 and TSH (Figure 2). At the same time, a higher number of referrals were made in the winter than in the summer, despite the fact that fewer specimens were submitted for screening from January to March than from July to September (Table 1). There were no significant differences in the number of confirmed cases between these two periods (Table 1). Rezaeian et al. [20] reported an increased incidence of CH during the summer months in the Hamadan Province of Iran. Other groups have also reported that the incidence of CH varies with the seasons [21,22]. However, climate differences have not been observed in all studies [23,24,25]. In NYS, a significant seasonal difference is not observed in the incidence of CH (Table 1).

NBS for CH leads to a significant number of false-positive results, which causes anxiety in parents and additional work and cost for the health care system. From 2008 to 2017, the PPV for screening during January–March was 14.7% but the value was 20.6% from July–September, reflecting the higher false-positive rate during the colder season. The overall PPV from 2008 to 2017 was 18.7%, and borderline results led to a recall rate of 1.57%. Similarly, other NBS programs have low PPV screening results for CH [26]. The main reasons for the low PPV are the variabilities seen in T4 and TSH values mainly due to the TSH surge at birth and premature and sick infants having lower T4 values that may not stabilize for weeks [27,28]. To reduce the recall rates, age-adjusted cut-offs for TSH and T4 are recommended. In addition, routine repeat screening for all LBW, premature, ill and newborns admitted to NICU is recommended at 2–4 weeks of age and/or at discharge [1]. To avoid missing cases with delayed TSH rise in preterm newborns, McGrath et al. [27] recommend repeat screening at 1, 2, and 4 weeks and term-corrected gestational age or discharge.

In NYS, prior to March 2020, T4 was the primary screen for CH. However, based on the NYS algorithm, most referrals were a result of elevated TSH values rather than low T4 values (Figure 1). In winter months, when measured T4 and TSH values were higher, the elevated TSH values caused a larger number of false-positive cases (Table 1). In the summer, when measured TSH values were lower, there were fewer referrals because fewer specimens reached the referral-level cut-off. However, because of the lower measured T4 values, some additional specimens were referred based on their low T4 value. There were more false-positives based on elevated TSH results in the winter than due to lower T4 values in the summer because most CH referrals in our program are based on elevated TSH results. This explains why our total false-positive cases are higher in the winter but our false-positive cases due to low T4 values are higher in the summer.

The relationship between temperature and the stability of markers in dried blood spots (DBS) has been studied by many investigators [29,30,31,32]. Lando et al. [32] reported that neonatal screening of dried blood samples kept at 4–8 °C are reliable for repeating hormonal measurements (including T4 and TSH) for up to three years. However, TSH was shown to experience significant degradation at 37 °C and low humidity [31]. In addition, it was shown that TSH degradation at high humidity was more than 3 times greater than its degradation at low humidity after a month of storage at 37 °C, and T4 degradation at high humidity was more than 10 times greater than its degradation at low humidity [31]. One study reported that TSH was relatively stable for one month when samples were exposed to high humidity or temperatures up to 37 °C [30]. The same study showed that T4 values decreased significantly after four days of suboptimal conditions [30]. Coombes et al. [29] have shown that TSH is stable in DBS for at least 30 days when stored at room temperature, 4 °C, or −20 °C but lose 16% activity when exposed to 37 °C for 6–8 days [29]. In our study, the lower measured T4 and TSH values in the warmer months could result from destabilization of the analyte and/or noneluting blood spot due to deterioration of sample caused by the high ambient temperatures or the high humidity to which NBS cards are exposed during transportation to the NBS laboratory.

Other theories put forward by researchers for elevated TSH during the winter are decreased maternal consumption of iodine-rich foods or lower maternal vitamin D during winter months and lower temperatures stimulating fetal pituitary to produce more TSH in the winter [33,34,35].

Setting aside seasonal variations, there was also a slight increase in daily mean T4 and TSH values over time (Figure 2a,b). One reason for the increase in daily mean TSH is that starting in 2013, the NBS program made a concerted effort to encourage hospitals to collect and submit specimens from neonatal intensive care unit babies prior to transfusion and therefore submission of specimens collected from infants <24 h of age increased over time. Additionally, in 2015 in the US, there was an initiative to set timeliness goals to achieve the earliest diagnosis and intervention for infants with disorders identified through NBS, which led to a decrease in age of collection [36]. In NYS, yearly T4 mean values were relatively constant from 2008 to 2014 but increased in 2015 (Figure 2b). The increase is observed across all categories of specimens irrespective of weight or age at collection. Therefore, fluctuations in the numbers of these specimens did not lead to the increase in mean T4 values. No known changes were made to instrumentation, specimen submission, or specimen handling at this time. We suspect that a change was made to the kit, although the manufacturer, when contacted, did not report any changes. From 2008 to 2017, the laboratory changed T4 kits approximately every 3–6 months using a total of 34 different kit lots. The evaluation of the quality control materials indicates that subsequent to some T4 kit lot changes (primarily on 27 April, 2015), the measured values of the control material increased. As expected, seasonal changes did not affect the values of quality control materials as kit performance was not affected. The absence of changes in values of quality control material with the exception of rare instances that coincided with a kit lot change supports the hypothesis that external factors (e.g., influence of heat and humidity on dried blood spots) resulted in the observed seasonal variation.

It should be noted that in winter, a slightly higher proportion of specimens were submitted to our program from LBW infants and from >14-day-old infants. Due to the immaturity of the hypothalamic–pituitary axis, false-positive CH results are more common in LBW infants as their T4 values are low [37]. In addition, older infants have lower T4 values [38]. Not only did LBW and older age not contribute to the elevated mean daily T4 observed in winter, but in the absence of seasonal variation, the expectation would be that daily mean T4 values would be lower in the winter due to the higher proportion of specimens from these categories of infants.

Our data show that seasonal variations affect measured T4 and TSH values and consequently the CH recall rate. Additionally, kit alterations should be closely monitored as unanticipated changes in values may also occur. NBS programs using fixed cut-off values for referring infants should be aware of any variation causing an increase in TSH or a decrease in T4 values, because higher TSH or lower T4 values may lead to an increase in the number of false-positive screens. In addition, increasing the TSH cut-off or lowering the T4 cut-off could potentially increase false-negative screens in the summer.

## Figures and Tables

**Table 1:** Comparison between winter and summer for babies tested, referred, and false-positive cases from 2008 to 2017.

	Winter (January–March)	Summer (July–September)	*p*-Value
Total Babies Tested	590,553	640,863	<0.0001
Total Referrals	2033	1758	<0.0001
Total Confirmed CH	236	282	0.2663
Total False-Positive	1368	1088	<0.0001
Cases Closed as “Other” *	429	388	-
Incidence of CH	1 in 2502	1 in 2273	-
Referral Rate	1 in 290	1 in 365	-
False-Positive Rate	1 in 432	1 in 589	-
Positive Predictive Value (PPV)	14.7%	20.6%	<0.0001

* “Other” cases include lost to follow-up, deceased, thyroxine-binding globulin (TBG) deficiency, disease of other etiology, hypothyroxinemia of prematurity, hyperthyrotropinemia of prematurity, blocking maternal TSH receptor antibodies, and maternal medication.

## Supplementary Materials

The following are available online at https://www.mdpi.com/2409-515X/7/1/8/s1, Figure S1: Daily mean TSH values for all specimens excluding specimens collected <24 h after birth, from 2008 to 2017; Figure S2: Error plot for daily mean T4 value for 2014 plotted against ambient temperature. Note that the error centers around zero.
