# Supplementary figures and images for: The Impact of Seasonal Changes on Thyroxine and Thyroid-Stimulating Hormone in Newborns
Source: Int J Neonatal Screen. 2021 Feb 3;7(1):8. doi: 10.3390/ijns7010008 (PMC7930942; doi:10.3390/ijns7010008)

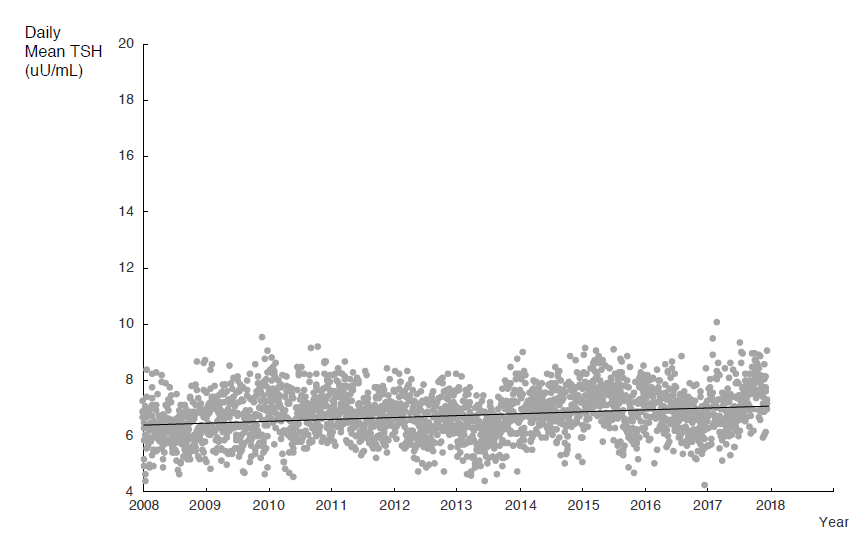

Supplement: Supplementary file 1 [file IJNS-07-00008-s001.zip › Supplemental Figure 1 - 2.1.21.PNG]

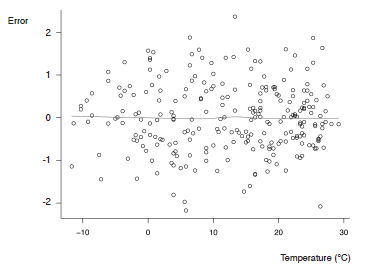

Supplement: Supplementary file 1 [file IJNS-07-00008-s001.zip › Supplementarl Figure 2.PNG]
